# Strongyloidiasis as a Focal Household Cluster of Soil‐Transmitted Helminthiasis in Rural Tropical Rainforest Southern Thailand: An Observational Study

**DOI:** 10.1155/sci5/6861451

**Published:** 2026-10-18

**Authors:** Attarat Pattanawongsa, Prasit Na-ek, Patthanasak Khammaneechan, Blego Sedionoto, Witthaya Anamnart

**Affiliations:** ^1^ School of Pharmacy Walailak University Nakhon Si Thammarat 80161 Thailand wu.ac.th; ^2^ Drug and Cosmetics Excellence Center Walailak University Nakhon Si Thammarat 80161 Thailand wu.ac.th; ^3^ Department of Medical Science School of Medicine Walailak University Nakhon Si Thammarat 80161 Thailand wu.ac.th; ^4^ Research Center in Tropical Pathobiology Walailak University Nakhon Si Thammarat 80161 Thailand wu.ac.th; ^5^ School of Public Health Walailak University Nakhon Si Thammarat 80161 Thailand wu.ac.th; ^6^ EC for PHR Walailak University Nakhon Si Thammarat 80161 Thailand wu.ac.th; ^7^ Department of Environmental Health Faculty of Public Health Mulawarman University Samarinda East Kalimantan Indonesia unmul.ac.id; ^8^ School of Allied Health Sciences Walailak University Nakhon Si Thammarat 80161 Thailand wu.ac.th

**Keywords:** close-contact transmission, hookworm infection, latrine, strongyloidiasis

## Abstract

**Background:**

In southern Thailand, characterized by high rainfall for 9 months each year (2340 mm annually), hookworm prevalence is consistently higher than that of *Strongyloides stercoralis*. Such wet climatic conditions may favor hookworm transmission while limiting *S. stercoralis*. Conversely, living in crowded, cluster settlements may enhance the transmission of *S. stercoralis*. We aimed to determine whether *S. stercoralis* exhibits a focal distribution pattern—often related to close contact within households or small communities—and whether this pattern distinctively differs from that of hookworm infection.

**Methods:**

We conducted a comparative observational study involving 346 of 418 village residents across eight designated subareas. Subareas 2, 4, 6, and 7 featured cluster settlements; Subareas 3 and 5 were diffuse low‐income settings; and Subareas 1 and 8 represented mixed‐density low‐income areas. Each subarea contained at least one household lacking latrines. Stool samples were collected and analyzed using agar plate culture and the modified formalin‐ether concentration technique to detect helminths. The spatial distributions of both infections were subsequently mapped and analyzed using spatial dispersion indices.

**Results:**

Of the eight subareas, six demonstrated a higher hookworm‐to‐*S. stercoralis* prevalence ratio, ranging from 1.59:1 to 10:1. Conversely, Subareas 2 and 8 exhibited near‐equivalent ratios, ranging from 1.38:1 to 1.75:1 (exact binomial *p* = 0.453, OR = 2.50 [discordant pairs = 7] and exact binomial *p* = 0.109, OR = 4.00 [discordant pairs = 10], respectively). Subareas 3 and 5 showed the strongest dominance of hookworm over *S. stercoralis*, at 10:1 and 7.7:1, respectively. Geographical and inequality indices revealed that *S. stercoralis* clustered focally across all subareas except 3 and 5, whereas hookworm infections were more broadly and uniformly distributed.

**Conclusion:**

In southern Thailand, strongyloidiasis appears to cluster within households, supporting the hypothesis of close‐contact transmission, whereas hookworm infection is more widely distributed. These spatial differences suggest distinct, tailored targeting strategies are necessary for hookworm control and *S. stercoralis* interventions.

## 1. Introduction

Hookworm, *Ascaris lumbricoides*, *Trichuris trichiura*, and *Strongyloides stercoralis* are soil‐transmitted helminths. In 2010, hookworm caused the most years lived with disability (YLDs) [1]. Hookworm infects about 438 million people globally [1], while *S. stercoralis* infects between 30 and 100 million [2]. Because both hookworm and *S. stercoralis* third‐stage larvae (L3) infect humans via skin penetration, one might expect a 1:1 prevalence ratio between hookworm infection and strongyloidiasis when using agar plate culture (APC) or the Baermann method. However, their prevalence varies widely by region. In Thailand, with annual rainfall between 1361 and 1781 mm [3], the observed ratio ranged from 0.43:1 to 2.3:1 [4–6]. In southern Thailand, where rainfall is 2340 mm [3], the hookworm ratio was much higher, ranging from 2.5:1 to 13.1:1 [7–9]. In African regions such as Tanzania, Ethiopia, the Ivory Coast, and Ghana (rainfall 1365–1848 mm [3]), the ratio also favored hookworm, ranging from 1.63:1 to 5.4:1 [10–13]. Conversely, Angola and Mozambique (rainfall 219–800 mm [3]) showed a lower ratio, from 0.48:1 to 0.83:1 [14, 15], indicating that strongyloidiasis was relatively more prevalent there. This variation may reflect factors such as sanitation, mass drug administration, and climate. In regions with prolonged rainfall, the prevalence of hookworm infection is consistently higher than that of strongyloidiasis. In contrast, in lower rainfall regions, the prevalence of strongyloidiasis is higher than that of hookworm infection. Hookworm and *S. stercoralis* need enough moisture for growth and development. However, excessive rainwater may affect *S. stercoralis* growth and development more than hookworm [16].

In Southeast Asia, especially in the rice‐farming regions of northeastern Thailand, Laos PDR, and Cambodia, where annual rainfall ranges from 1361 to 1781 mm [3], strongyloidiasis is widespread among farmers. More than 50% of *S. stercoralis*‐positive participants were farmers [17–21]. Because water is essential for rice cultivation in these regions, rice must be grown during the rainy season. These farmers often work together all day during the rice cultivation season. Temporary housing in the rice field lacks latrines, so farmers must defecate in the moist field. This may create conditions in which *S. stercoralis* first‐stage larvae (L1) can develop into free‐living males and females. These mate and produce a large number of L3, and farmers can frequently become infected with *S. stercoralis* L3 in these environments. This close‐contact behavior may help explain the high prevalence of infection among farmers in these regions. Latrines could prevent the transmission of strongyloidiasis [22] because *S. stercoralis* L1 cannot survive in septic tanks [23]. However, their use is not feasible due to the farmers’ occupation.

In combination with the local climate, a way of life that fosters close contact among people in isolated, close‐knit communities can characterize farmers’ behavior, increasing the likelihood of clustered strongyloidiasis. By comparing hookworm and strongyloidiasis cases within villages in Nakhon Si Thammarat, Thailand, with an annual rainfall of 2340 mm [3], this study aims to determine (1) whether *S. stercoralis* tends to demonstrate clustered, focal transmission related to close contact within households or small communities and (2) whether hookworm has widespread transmission.

## 2. Materials and Methods

### 2.1. Study Area

In 2010, the study was conducted in southern Thailand, 8 km south of Walailak University. It covered specific subareas in three villages: 5 from Village 11 and 1 from Village 13 (both in Mokhalan Subdistrict), and 2 from Village 6 (Phothong Subdistrict), all within Thasala District. In total, the study area is divided into eight subareas: Subarea 1 in Village 13, Subareas 2–6 in Village 11, and Subareas 7‐8 in Village 6. Some subareas (2, 4, 6, and 7) are geographically isolated (Figures 1 and 2). Households in these areas often consist of extended families or relatives living close together. There are 114 households with 418 members altogether, distributed as follows: Subarea 1, 35 households/124 people; Subarea 2, 3/15; Subarea 3, 31/95; Subarea 4, 3/14; Subarea 5, 25/102; Subarea 6, 8/35; Subarea 7, 2/13; and Subarea 8, 7/20. The number of households without latrines is 4, 3, 2, 1, 2, 2, 1, and 1 for Subareas 1 through 8, respectively. Subareas 2, 4, 6, and 7 are cluster settlements. Conversely, Subareas 3 and 5 are diffuse low‐income areas, while Subareas 1 and 8 are mixed‐density low‐income areas, a combination of the two.

**FIGURE 1:**
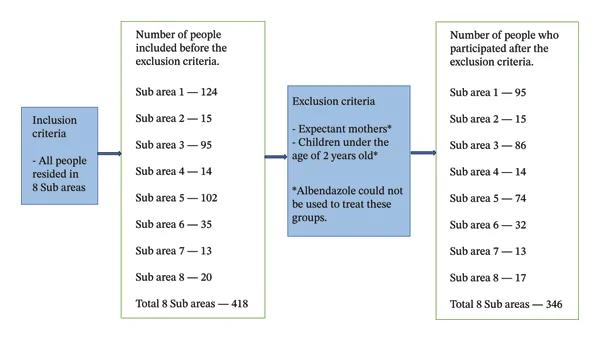
The number of included participants after applying the exclusion criteria.

**FIGURE 2:**
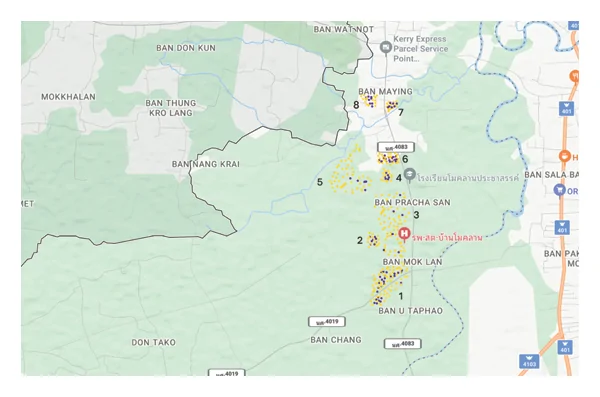
Distribution of strongyloidiasis (dark purple) among widespread hookworm infection (dark yellow) in Subareas 1–8 (1, 2, 3, 4, 5, 6, 7, and 8). The map was downloaded from Microsoft Bing Maps. Microsoft product screenshots reprinted with permission from Microsoft Corporation.

### 2.2. Study Design and Sample Size

Due to the uncertain population sizes in the three villages, the sample size was calculated using the formula: *n* = *z* ^2^ *p*(1−*p*)/*e* ^2^, where *n* = sample size, *z* = 1.96, *e* = 0.05, and the prevalence (p) of strongyloidiasis was estimated in 2008 at 28.7% (0.287) [7]. Purposive sampling was used to include most residents of the 8 subareas. Participation in this study was voluntary. The following were excluded: (1) expectant mothers and (2) children under 2 years old. These groups were excluded because, according to participant information, a single or 3 consecutive doses of 400 mg albendazole would be used to treat hookworm‐ or *S. stercoralis*‐positive participants, and albendazole could not be used for these two groups.

An observational comparative study was conducted in 346 out of 418 household members (82.8%). The study included 95 (76.6%) from Subarea 1, 15 (100%) from Subarea 2, 86 (90.5%) from Subarea 3, 14 (100%) from Subarea 4, 74 (72.5%) from Subarea 5, 32 (91.4%) from Subarea 6, 13 (100%) from Subarea 7, and 17 (85%) from Subarea 8. After stool examination, we mapped the households with positive cases for *S. stercoralis* and hookworm to visualize their distribution patterns within the villages.

### 2.3. Stool Samples

We collected stool from 346 household members in 114 households. Stools were collected in the morning into clean plastic containers. They were then sent to the laboratory at Walailak University. We examined the stool using a modified formalin‐ether concentration technique (MFECT) and APC within 2 h of defecation.

### 2.4. Laboratory Procedure

MFECT was performed as follows [24]. Two grams of stool were suspended in 8 mL of NSS in a 15‐mL plastic tube. The suspension was strained through two wire meshes—a coarse one on top and a fine one on the funnel—into another tube. The strained suspension was centrifuged at 700 × g for 5 min. After decanting the supernatant, the volume was adjusted to 7 mL with 10% formalin. Then, 3 mL of diethyl ether was added, and the mixture was mixed well. The suspension was immediately centrifuged at 700 × g for 5 min. The debris plug was loosened and decanted. The sediment was adjusted to 0.75–1 mL (35–40 drops) with 10% formalin. Two drops were examined to count eggs or larvae, and the result was halved. The mean number of eggs was multiplied by 35–40, divided by 2, and reported as eggs or larvae per gram of stool (EPG or LPG). Negative results were reported after the entire sediment was examined.

APC was performed as follows [25]: 3 g of stool was placed at the center of the nutrient agar plate. The plate was incubated at room temperature (26°C–32°C) for 6 days. On Day 6, 10 mL of 10% formalin was added to the agar surface of the positive APC sample and left to stand for 5 min. The formalin suspension was transferred back to a 15‐mL tube and centrifuged at 700 × g for 5 min. After the supernatant was decanted, we examined *S. stercoralis* or hookworm L3 in the pellet suspension.

Hookworm L3 had a short filariform esophagus and a pointed tail, while *S. stercoralis* L3 had a long esophagus about half of the body length and a notched tail. Furthermore, *S. stercoralis* L3 were slim, but hookworm L3 were fat. If negative, the plate was checked for tracks from larval crawling after 6 days of incubation.

### 2.5. Statistical Analyses

The spatial distribution of hookworm‐positive and *S. stercoralis*‐positive cases was mapped using base maps obtained from Microsoft Bing Maps. The McNemar test was employed to assess differences in the proportions of hookworm mono‐infection and *S. stercoralis* mono‐infection across the subareas. However, for Subareas 2, 4, 7, and 8, the exact binomial test was applied because the total number of discordant pairs (the sum of discordant cells) was less than 25.

The McNemar test was selected because it is specifically designed for binary paired/matched data. In this study, the comparisons were made on a dependent/matched population (individual household members tested simultaneously for both parasites) to evaluate the relative proportion of hookworm mono‐infection against *S. stercoralis* mono‐infection within each subarea. Furthermore, to maintain statistical rigor given our sample size limitations in specific sectors, we incorporated the exact binomial test as a corrective measure.

For the paired/matched binary data analyzed via the McNemar test, the matched odds ratio (OR) was calculated as the ratio of the discordant cells using the standard maximum likelihood estimation: OR = *b*/*c*, where *b* represents the number of individuals with hookworm mono‐infection and *c* represents the number of individuals with *S. stercoralis* mono‐infection. The corresponding 95% confidence intervals (95% CI) for the paired OR were computed by first calculating the standard error of the natural logarithm of the OR:

(1) SE In OR=1b+1c.

The upper and lower bounds of the 95% CI were then calculated by exponentiating the log‐transformed bounds using the Wald/Woolf method: 95% CI = *e* ^ln(OR)±1.96·SE(ln OR)^. For the subareas analyzed via the exact binomial test due to small discordant cell counts (*b* + *c* < 25), the exact 95% CI for the paired proportions were calculated based on the cumulative binomial distribution.

To determine the spatial dispersion patterns, the mean, standard deviation (SD), variance, and variance‐to‐mean ratio (VMR) of the parasite prevalence across the eight subareas were calculated. A chi‐square test was used to evaluate whether the observed VMR values deviated significantly from a random spatial distribution (VMR = 1).

Furthermore, the Gini coefficient (*G*) was computed in Microsoft Excel to evaluate the degree of distribution inequality across the subareas using Gini’s mean difference formula:

(2) G=∑i=1 to n∑j=1 to n│xi−xj│ 2∗n2∗x− ,

where *x*_*i*_ and *x*_*j*_ represent the parasite prevalence in each subarea, │*x*_*i*_ − *x*_*j*_│ is the absolute difference between all possible pairs of subarea prevalence, *n* is the total number of subareas (*n* = 8), and x̄ is the mean prevalence across all subareas.

The unit of analysis was the individual household member (*n* = 346) for the McNemar and exact binomial tests, and the subarea (*n* = 8) for the VMR and Gini coefficient calculations.

For all tests, a *p* value of < 0.05 was considered statistically significant. Except for the Gini coefficient calculations, all statistical analyses were performed using IBM SPSS Statistics for Windows, Version 29.0 (IBM Corp., Armonk, NY, USA).

## 3. Results

Figure 1 shows the total number of people included in the study area and the number of participants after applying the exclusion criteria.

The ratio of individuals with hookworm infection to those with strongyloidiasis ranged from 1.38:1 to 10:1. Although the prevalence of strongyloidiasis was consistently lower than that of hookworm infection, the prevalence ratio in Subareas 2 (1.38:1) and 8 (1.75:1) was not significantly different (exact binomial *p* = 0.453, OR = 2.50 [discordant pairs = 7] and exact binomial *p* = 0.109, OR = 4.00 [Discordant pairs = 10], respectively). In isolated Subareas 2, 4, 6, and 7, which are characterized by cluster settlements, the prevalence ratio ranged from 1.38:1 to 2.3:1. In contrast, in Subareas 3 and 5, which feature diffuse low‐income settings, the prevalence ratio ranged from 7.7:1 to 10:1, while in Subareas 1 and 8, characterized by mixed‐density low‐income areas, the ratio ranged from 1.75:1 to 2.56:1 (Table 1).

**TABLE 1:** The rate of hookworm infection to strongyloidiasis ratio in 8 subareas of the studied village before the drought.

Subarea no.	No. of house holds	No. of household members	No. of household members examined (%)	No. of households with no latrine^a^	No. of hookworm positive (%)	No. of *S. stercoralis* positive (%)	Prevalence ratio of hookworm to *S. stercoralis*	McNemar/exact binomial test statistics (matched OR [95% CI])
1	35	124	95 (76.6)	4	69 (72.6)	27 (28.4)	2.56:1	*χ* ^2^(1) = 35.02, *p* < 0.001; OR = 15.00 (95% CI: 4.66–48.27)
2	3	15	15 (100)	3	11 (73.3)	8 (53.3)	1.38:1	Exact binomial *p* = 0.453; OR = 2.50 (Discordant pairs = 7)
3	31	95	86 (90.5)	2	60 (69.8)	6 (7.0)	10:1	*χ* ^2^(1) = 48.43, *p* < 0.001; OR = 28.00 (95% CI: 6.83–114.74)
4	3	14	14 (100)	1	14 (100)	6 (42.8)	2.3:1	Exact binomial *p* = 0.008 (Discordant pairs = 8)
5	25	102	74 (72.5)	2	46 (62.2)	6 (8.1)	7.7:1	*χ* ^2^(1) = 33.07, *p* < 0.001; OR = 21.00 (95% CI: 5.08–86.75
6	8	35	32 (91.4)	2	27 (84.4)	17 (53.1)	1.59:1	*χ* ^2^(1) = 5.06, *p* = 0.024; OR = 4.33 (95% CI: 1.23–15.21)
7	2	13	13 (100)	1	13 (100)	7 (53.8)	1.86:1	Exact binomial *p* = 0.031 (discordant pairs = 6)
8	7	20	17 (85)	1	14 (82.4)	8 (47.1)	1.75:1	Exact binomial *p* = 0.109; OR = 4.00 (discordant pairs = 10)
Total	114	418	346 (82.8)	16	254 (73.4)	85 (24.6)	3:1	*χ* ^2^(1) = 141.83, *p* < 0.001; OR = 12.27 (95% CI: 7.25–20.76)

^a^All households had at least one *S. stercoralis*‐positive household member, and at least one hookworm‐positive member.

Compared to strongyloidiasis, hookworm infection showed a widespread distribution across all eight subareas of the village with a mean prevalence of 80.6% (variance = 192), yielding a VMR of 2.4 (*χ* ^2^(7) = 16.68, *p* = 0.020) and a Gini coefficient of 0.089. In contrast, strongyloidiasis was limited to clustered areas within the hookworm‐infected regions (mean = 36.7%, variance = 393), characterized by a markedly high VMR of 10.7 (*χ* ^2^(7) = 75.04, *p* < 0.001) and a Gini coefficient of 0.269 (Table 2). Specifically, strongyloidiasis formed distinct infection clusters in Subareas 1, 2, 4, 6, 7, and 8, rather than being uniformly distributed throughout the entire village (Figure 2). Furthermore, strongyloidiasis cases exhibited a restricted distribution in Subareas 3 and 5, despite the presence of *S. stercoralis*‐infected individuals residing in households without latrines.

**TABLE 2:** Spatial distribution and inequality indices of hookworm and *S. stercoralis* prevalence by subareas.

Subarea no.	Prevalence of hookworm infection	Prevalence of strongyloidiasis
1	72.6	28.4
2	73.3	53.3
3	69.8	7.0
4	100	42.8
5	62.2	8.1
6	84.4	53.1
7	100	53.8
8	82.4	47.1
Mean (SD)	80.6 (13.9)	36.7 (19.8)
Variance	192	393
VMR	2.4 (*χ* ^2^[7] = 16.68, *p* = 0.020)	10.7 (*χ* ^2^[7] = 75.04, *p* < 0.001)
Gini coefficient	0.089	0.269

## 4. Discussion

In the present study, strongyloidiasis demonstrated a focal pattern, whereas hookworm infection was widespread. The focal or clustered distribution of strongyloidiasis, characterized by a markedly high VMR of 10.7 (*χ* ^2^(7) = 75.04, *p* < 0.001) and a Gini coefficient of 0.269 (Table 2), indicated that the prevalence of strongyloidiasis was unevenly distributed and substantially exceeded the overall average of 24.6% (Table 1) in specific localities. Specifically, focal clustering was observed in Subareas 2, 4, 6, and 7, which feature clustered settlements, as well as Subareas 1 and 8, which are located in mixed‐density, low‐income areas.

This clustered distribution of strongyloidiasis appears to be associated with areas where people live in crowded conditions, potentially due to several interrelated factors: (1) Outdoor defecation practices in open holes may facilitate the life cycles of both hookworm and *S. stercoralis*. (2) Although *S. stercoralis* L3 larvae survive for up to 10 days—which is four times shorter than the survival span of hookworm [16]—this biological disadvantage may be mitigated by crowded living conditions that shorten the distance to a new host. (3) While both parasites are highly susceptible to desiccation [26], during dry seasons or low‐rainfall periods, *S. stercoralis* L3 may have higher relative opportunities to infect new hosts than hookworm L3 due to its shorter production period. In tropical rainforest climates, 5%–10% of *S. stercoralis* L1 larvae can develop into infective L3 within a single day, compared to approximately 6 days required for hookworm [27, 28].

Conversely, in Subareas 3 and 5, which are characterized by diffuse, low‐income settings where residences are scattered, *S. stercoralis* did not exhibit a clustered distribution. This implies that scattered living arrangements may naturally disrupt the transmission intensity of this parasite. Furthermore, heavy rainfall during the wet season may dilute or wash away environmental stages. While rainwater carrying *S. stercoralis* stages (except L3) may reduce their viability and overall environmental numbers [29], rainwater‐diluted stool might not affect hookworm L1 development. Hookworm L1 larvae require organic matter from bacteria, microorganisms, or stool [26] and can freely develop either in diluted stool or directly in the soil [16, 26]. In contrast, *S. stercoralis* L1 larvae may require not only bacteria but also specific concentrations of soluble stool components [16, 29], meaning they likely need to grow in stool diluted to no more than 1:160 [29] and fail to develop freely in ordinary soil [16, 29]. If the environmental dilution exceeds 1:160, or if the stool remains submerged under rainwater for more than 12 h, free‐living generations fail to successfully produce L3 larvae [30].

Additionally, while rainwater can transport the L3 larvae of both parasites from one area to another, hookworm L3 survives for up to 40 days, whereas *S. stercoralis* L3 survives for only 10 days [16, 26]. Hence, heavy rainfall and environmental washing may favor hookworm dispersion while suppressing *S. stercoralis*, potentially contributing to the higher hookworm‐to‐strongyloidiasis prevalence ratios observed in Subareas 3 and 5. Notably, although hookworm infection was widespread across all subareas, its prevalence in Subareas 3 and 5 was lower than in the other six subareas, suggesting that crowded conditions may enhance the transmission dynamics of both hookworm and *S. stercoralis*.

The use of septic tank sludge as fertilizer around households may also influence these dynamics. *S. stercoralis* L1 larvae fail to develop under anaerobic conditions like those in a septic tank [23]. However, hookworm eggs can undergo cleavage arrest and survive for up to 45 days. When exposed to air after removal from sludge, these hookworm eggs can resume development and become infective L3 within 6 days [28]. Thus, the application of septic tank sludge as fertilizer may actively promote the spread of hookworms but not *S. stercoralis* [23].

Because the infective L3 larvae of both hookworm and *S. stercoralis* infect humans via skin penetration, their theoretical prevalence ratio could be expected to approach 1:1 under identical exposures. In the present study, a higher prevalence ratio favoring hookworm was found in six out of eight subareas, ranging from 1.59:1 to 10:1. However, Subareas 2 and 8 showed near‐equivalent ratios ranging from 1.38:1 to 1.75:1. This near‐equal ratio may be associated with wet areas featuring clustered settlements where overcrowding is prevalent, suggesting that close‐contact living conditions can increase the transmission efficiency of *S. stercoralis* even when climatic conditions generally favor hookworms. Factors beyond the annual rainfall of 2340 mm, including household sanitation and personal hygiene practices, likely play a combined role in shaping these varied prevalence ratios [7].

The clustered distribution of strongyloidiasis in Subareas 2, 4, 6, and 7, where people lived in crowded cluster settlements, may help contextualize the widespread infection patterns often observed among farmers in Northeastern Thailand, Laos, and Cambodia [17–21]. In these subareas, residents live in isolated yet highly dense clustered settlements. Similarly, farmers—who represent a population at high risk for strongyloidiasis—frequently work in close proximity during rice cultivation. Defecation in agricultural fields near temporary housing provides optimal microenvironmental conditions for the growth and development of *S. stercoralis*. Consequently, farmers are exposed to *S. stercoralis* L3 within localized, focal clusters. When participants are randomly sampled across large agricultural regions, the cumulative effect of these small, focally distributed clusters can lead to a high overall prevalence among farmers, as seen in previous studies where more than 50% of randomly sampled participants were farmers [17–21]. Thus, occupations or living conditions that involve close contact appear to enhance the transmission of strongyloidiasis in areas where moist conditions are optimal.

## 5. Conclusion

In a wet region, strongyloidiasis shows localized household clustering, supporting the hypothesis of close‐contact transmission, while hookworm infection is more widely distributed. These spatial differences support the implementation of distinct targeting strategies for hookworm control and strongyloidiasis interventions.

## Author Contributions

Attarat Pattanawongsa designed and performed experiments, analyzed the data, and wrote the paper. Prasit Na‐ek designed experiments, analyzed the data, and wrote the paper. Patthanasak Khammaneechan conceived the study, designed experiments, analyzed the data, and wrote the paper. Blego Sedionoto analyzed the data and wrote the paper. Witthaya Anamnart conceived the study and writing–original draft.

## Funding

No funding was received for this manuscript.

## Disclosure

All authors discussed the results and reviewed the manuscript.

## Ethics Statement

Ethical approval was granted by the Ethics Committee in Human Research at Walailak University, Thailand, under approval number 10/065. Participants reviewed the information sheet and provided written informed consent to join the study. Guardians gave written consent for participants under 18.

## Conflicts of Interest

The authors declare no conflicts of interest.

## Data Availability

Original contributions from this study are available in the article. For further inquiries, please contact the corresponding author.
